# Endocytic Adaptor Protein HIP1R Controls Intracellular Trafficking of Epidermal Growth Factor Receptor in Neuronal Dendritic Development

**DOI:** 10.3389/fnmol.2018.00447

**Published:** 2018-12-06

**Authors:** Qian Yang, Lin Peng, Yu Wu, Yanan Li, Ling Wang, Jian-hong Luo, Junyu Xu

**Affiliations:** ^1^Department of Neurobiology, Institute of Neuroscience, NHC and CAMS Key Laboratory of Medical Neurobiology, Zhejiang University School of Medicine, Hangzhou, China; ^2^Department of Psychiatry, Jining Medical University, Jining, China; ^3^Department of Anesthesiology, Sir Run Run Shaw Hospital, Zhejiang University School of Medicine, Hangzhou, China

**Keywords:** HIP1R, neurite initiation, dendritic branching, EGFR, endocytosis, signaling pathway

## Abstract

Huntington-interacting protein 1-related protein (HIP1R) was identified on the basis of its structural homology with HIP1. Based on its domain structure, HIP1R is a putative endocytosis-related protein. Our previous study had shown that knockdown of HIP1R induces a dramatic decrease of dendritic growth and branching in cultured rat hippocampal neurons. However, the underlying mechanism remains elucidative. In this study, we found that knockdown of HIP1R impaired the endocytosis of activated epidermal growth factor receptor (EGFR) and the consequent activation of the downstream ERK and Akt proteins. Meanwhile, it blocked the EGF-induced dendritic outgrowth. We also showed that the HIP1R fragment, amino acids 633–822 (HIP1R_633–822_), interacted with EGFR and revealed a dominant negative effect in disrupting the HIP1R-EGFR interaction-mediated neuronal development. Collectively, these results reveal a novel mechanism that HIP1R plays a critical role in neurite initiation and dendritic branching in cultured hippocampal neurons via mediating the endocytosis of EGFR and downstream signaling.

## Introduction

The growth and branching of dendrites are crucial for proper neuronal communication and nervous system function. They are controlled by both external signals and intrinsic genetic programs, such as secreted proteins and cell surface receptors (Lein et al., 1995; McAllister et al., 1997; Hoogenraad et al., 2005; Rosso et al., 2005; Dimitrova et al., 2008), cytoskeletal regulators (Luo, 2002; Scott et al., 2003; Newey et al., 2005), motor proteins (Hoogenraad et al., 2005; Wynshaw-Boris, 2007; Zheng et al., 2008), and cell adhesion molecules which are involved in dendrite morphogenesis (Fuerst et al., 2008, 2009). In addition, endocytic pathways also contribute to dendritic growth by modulating signaling pathways in response to cell surface receptor internalization (Ullrich et al., 1996; Satoh et al., 2008; Lazo et al., 2013).

Clathrin-mediated endocytosis is one of the major classes of endocytosis (Bonifacino and Lippincott-Schwartz, 2003). During endocytosis, clathrin triskelions associate with endocytic adaptor proteins to assemble clathrin-coated pits (Owen et al., 2004). These adaptor proteins perform as a bridge, which simultaneously bind to clathrin and to transmembrane proteins and/or phospholipids, and also interact with each other and other components of the clathrin-coated pits (Kirchhausen, 2000). Huntingtin-interacting protein 1-related protein (HIP1R) is identified as one of endocytic adaptor proteins and belongs to Sla2/HIP1 family (Engqvist-Goldstein et al., 1999; Chopra et al., 2000). It is comprised of an AP180 N-terminal homology (ANTH) domain that binds to inositol phospholipids (Metzler et al., 2001; Hyun et al., 2004b; Legendre-Guillemin et al., 2004), a central coiled-coil domain for clathrin light-chain binding (Brett et al., 2002; Chen and Brodsky, 2005; Legendre-Guillemin et al., 2005), and an F-actin-binding region (THATCH domain) in the C-terminus (Legendre-Guillemin et al., 2002; Brett et al., 2006). In addition, HIP1R also binds to other endocytic related proteins such as cortactin (Le Clainche et al., 2007), Epsin (Brady et al., 2010), and Cbl-interacting protein of 85 kDa (CIN85; Kowanetz et al., 2004).

Several studies in proliferating cell lines showed that HIP1R is involved in events associated with epidermal growth factor receptor (EGFR). In HEK293T cells, overexpression of HIP1 or HIP1R stabilizes the pools of growth factor receptors, such as EGFR and platelet-derived growth factor-β receptor (PDGFβR; Hyun et al., 2004b). In addition, HIP1R associates with EGFR independent of its lipid-, clathrin-, or actin-binding domains (Bradley et al., 2007). While in HeLa cells, HIP1R knockdown stabilizes clathrin-coated structures and the endocytic cargos including EGF (Engqvist-Goldstein et al., 2004). These studies suggest that HIP1R may contribute to the endocytosis of EGFR. However, the relationship of these two proteins in the central nervous system (CNS) is still unknown.

In CNS, EGF and EGFR are expressed in brain regions including cortex, hippocampus and cerebellum during development and also in adulthood (Gómez-Pinilla et al., 1988; Lazar and Blum, 1992; Tucker et al., 1993; Misumi and Kawano, 1998). In neuronal cells, the activation of EGFR could promote neurite outgrowth. For instances, suppressor of cytokine signaling two induces phosphorylation of EGFR in PC12 cells and cortical neurons and leads to neurite outgrowth (Goldshmit et al., 2004a,b). Mevastatin induces neurite outgrowth via EGFR in Neuroblastoma Cells (Evangelopoulos et al., 2009). Moreover, Tissue kallikrein promotes neurite outgrowth via EGFR, flotillin-2 and ERK1/2 signaling pathway in cultured primary neurons and human SH-SY5Y cells (Lu et al., 2014). Our previous work showed that HIP1R deficiency impairs dendritic branching and spine formation in cultured hippocampal neurons, while overexpressed HIP1R increased dendrite numbers and spine density, demonstrating a critical role of HIP1R in neuronal morphology shaping. Taken together, HIP1R and EGFR are both involved in and may work together to induce neuron development.

Here we used RNAi and pharmacological treatment assays to investigate the role of HIP1R and EGFR in neuron development, and further characterized the underlying mechanisms. We found out that HIP1R interacts with EGFR and induces EGFR endocytosis to participate in dendritic development.

## Materials and Methods

### Animals

Pregnant Sprague-Dawley rats were bought from Shanghai SLAC Laboratory Animal Co., Ltd. and were housed under a 12:12 h light/dark cycle (lights on at 7:00 am) and constant temperature with free access to food and water in the laboratory animal center of Zhejiang University.

This study was carried out in accordance with the principles of the Basel Declaration and recommendations of US National Institutes of Health Guidelines for the Use and Care of Laboratory Animals. The protocol was approved by the Animal Advisory Committee at Zhejiang University.

### RNAi and DNA Constructs

The rat HIP1R RNAi construct was generated as previously described (Peng et al., 2017).

Mouse HIP1R homologous cDNA plasmids were used for overexpression experiments. Wild-type full-length cDNA of mouse HIP1R (NM_145070.3) was obtained and constructed into pEGFP-C3 vector as previously described (Peng et al., 2017). mRFP-tagged TrkB was a generous gift from Prof. Pietro De Camilli (Yale University School of Medicine). HIP1R_1–632_, HIP1R_633–822_ and HIP1R_823–1068_ were constructed into vector pGEX-4T-1 using BamHI and Xhol I enzyme cutting sites. Additionally, HIP1R_633–822_ was constructed into vector pEGFP-C3 using Xhol I and EcoR I enzyme cutting sites to obtain GFP-tagged HIP1R_633–822_. All these expression constructs were verified by sequencing and tested for normal expression by western blotting (WB).

### Cell Culture and Transfection

Dissociated neuronal cultures were prepared from embryonic day 17 Sprague-Dawley rat pups as described previously (Tang et al., 2015). Briefly, hippocampus tissues were dissected, dissociated and plated onto Poly-L-Lysine (Sigma) coated 35-mm dishes with or without glass coverslips in plating medium [Dulbecco modified Eagle medium (DMEM) containing 2 mM Glutamax-I and 10% fetal bovine serum (FBS)]. Four hours after plating, the plating medium was substituted by Neurobasal Media containing 2% B27 supplement, 2 mM Glutamax and 0.2% Penicillin-Streptomycin (all cell culture reagents for primary neuron culture were purchased from Gibco unless stated otherwise). Half of the medium was replaced by fresh medium by every 3–4 days. At day *in vitro* (DIV) 2–3, cytosine arabinofuranoside was added to a final concentration of 2.5 μM to prevent glial cell proliferation. For analysis of HIP1R subcellular localization, neurons were fixed 4–5 h after plating without substituting the plating medium. For early morphological analysis, neurons were electroporated using Amaxa Nucleofector Device II (Lonza, Valais, Switzerland), plated at 200–300 cells/mm^2^ with or without treatment, and fixed 4–6 h after plating for neurite initiation analysis or at DIV 6 for dendritic branching analysis. For biochemical analysis, neurons were plated at 800–1,000 cells/mm^2^ and harvested at DIV 6–8 or DIV 12 depending on the assays.

HeLa cells were a generous gift from Dr. Qi Miao (Second Affiliated Hospital of Zhejiang University School of Medicine). The cells were maintained in DMEM with 10% FBS. For Immunocytochemistry (ICC), cells were plated onto coverslips, plasmids were transfected into cells using Lipofectamine 2,000 following the manufacturer’s instructions.

### Antibodies

The following primary antibodies were used in the described experiments: HIP1R (612118, BD Biosciences Pharmingen, WB 1:1,000), HIP1R (AB9882, Millipore, ICC 1:1,000), EGFR (ab52894, Abcam, WB 1:1,000, Immunoprecipitation (IP) 4 μg), MAP2 (M9942, Sigma-Aldrich, ICC 1:1,000), early endosomal autoantigen 1 (EEA1; 610456, BD bioscience, ICC 1:500), GFP (homemade, IP 2 μg; WB 1:2,000), Phospho-EGF Receptor (Tyr-1068; #3777, Cell Signaling, WB 1:500), HIP1 (ab181238, Abcam, WB 1:1,000), ERK (4696S, Cell Signaling, WB 1:2,000), Phospho-ERK (Thr202/Tyr204; 4370S, Cell Signaling, WB 1:2,000), Akt (2966S, Cell Signaling, WB 1:1,000), Phospho-Akt (Ser473; 4060S, Cell Signaling, WB 1:1,000), β-actin (A5316, Sigma-Aldrich, WB 1:10,000), GAPDH (#2118, Cell Signaling, WB 1:5,000), and TrkB (07-225, Millipore, ICC 1:200 for surface and 1:500 for intracellular staining).

The secondary antibodies used for immunostaining were anti-rabbit Alexa Fluor488 (A11010, Invitrogen, 1:1,000), anti-mouse Alexa Fluor488 (A11001, Invitrogen, 1:1,000), anti-rabbit Alexa Fluor546 (A10040, Invitrogen, 1:1,000), anti-mouse Alexa Fluor546 (A10036, Invitrogen, 1:1,000) and anti-mouse Alexa Fluor633 (A21126, Invitrogen, 1:1,000). Phalloidin (Yeasen, Shanghai, China) was used to visualize the F-actin and mixed with the secondary antibodies and applied at a final concentration of 100 nM.

The secondary antibodies used for WB were goat anti-mouse IgG-HRP (31460, Pierce, 1:10,000) and goat anti-rabbit IgG-HRP (31420, Pierce, 1:10,000).

### Pharmacological Treatment

For analysis of early neuronal development, cultured neurons were incubated with recombinant human EGF (Novoprotein, Shanghai, China) or brain derived neurotrophic factor (BDNF; Novoprotein, Shanghai, China) at final concentrations of 50 ng/ml (EGF) or 25 ng/ml (BDNF) for 5–6 h, and fixed for neurite initiation analysis. Otherwise, one more dose of EGF or BDNF was added when the cultured medium was changed at DIV 3, and neurons were fixed at DIV 5–6 for dendritic branching analysis. Recombinant human EGF was added to cultured neurons and kept for 15 min before neurons being harvested for WB. For BIBX-1382 blockade experiments, coverslips in 24-well plates were pretreated with BIBX-1382 (MedChem Express, Princeton, NJ, USA) in a final concentration of 0.5 μM for 5 min before adding EGF. Alternatively, neurons were treated with BIBX-1382 only.

### EGF Internalization

Recombinant human EGF (0.5 μg/μl) was labeled with Alexa Fluor 647 Oligonucleotide Amine Labeling Kits (A20196, Invitrogen, 1 μg/μl) according to manufacturer’s instruction.

Neurons at DIV 8 with or without HIP1R knockdown were incubated with 200 ng/ml Alexa-647-conjugated EGF (Alexa^647^-EGF) for 20 min to allow for internalization. Non-internalized surface-bound Alexa^647^-EGF was then stripped by acid buffer (0.5 M NaCl and 0.2 M acetic acid) at 4°C for 2 min. Neurons were then fixed and subjected for ICC.

### Immunocytochemistry

Cultured hippocampal neurons at different developmental stages were fixed with 4% paraformaldehyde (diluted in PBS) for 15 min. After fixation, neurons were washed quickly with PBS and incubated with blocking buffer (0.2% Triton X-100, 5% BSA diluted in PBS) at room temperature for 1 h. Neurons were then incubated with primary antibodies diluted in blocking buffer for 1–2 h at room temperature and washed three times with PBS (10 min each time). Secondary antibodies and/or TRITC Phalloidin were applied for 1 h at room temperature. Finally, after washing three times with PBS, coverslips were mounted on microscope slides with ProLong Diamond Antifade Mountant (P36961, Invitrogen) and kept in a dark before image acquisition.

For the surface staining of TrkB, neurons plated on coverslips were rinsed three times in extracellular sodium (ECS; 145 mmol/L NaCl, 5 mmol/L KCl, 2 mmol/L CaCl_2_, 1 mmol/L MgCl_2_, 5 mmol/L glucose, 10 mmol/L sucrose, 5 mmol/L HEPES, pH 7.4), incubated in anti-TrkB primary antibody (1:200) diluted in ECS at 37°C for 10 min, rinsed three times in ECS quickly, and incubated with anti-rabbit Alexa Fluor-546 secondary antibody (1:500) for another 10 min. After rinsing in ECS for another three times, neurons were fixed by 4% paraformaldehyde and subjected for intracellular staining as described above.

Confocal images were acquired using the following confocal microscope: Fluoview FV1000 (Olympus) with a 20× NA 1.3 objective or 60× NA objective.

### Colocalization Analysis

Colocalization of EGFR with EEA1 was quantified as previously described (Tang et al., 2015). Briefly, analysis was carried out using MetaMorph software to generate a binary image of colocalized pixels from two separate channels. Images were thresholded to distinguish EEA1 and EGFR puncta based on immunofluorescence signals. Colocalization was established for pixels whose intensities were higher than threshold, and the data were plotted as the ratio of the integrated intensity from the two images. For each neuron, a 100–150 μm segment from each of three dendrites of similar thickness was selected for analysis.

### Biotinylation Assay

A biotinylation assay was performed as reported previously (Zhang et al., 2015). In general, high density cultured hippocampal neurons at DIV 12 were rinsed twice with buffer A (1× PBS with 0.5 mM MgCl_2_ and 1 mM CaCl_2_). Next, cells were incubated with 1 mg/ml sulfo-NHS-SS-biotin in buffer A for 30 min at 4°C, then washed twice (5 min each time) with a quenching agent (1 mM glycine in buffer A). Finally, after washing three times with buffer A, cells were lysed with RIPA buffer (50 mM Tris, pH 7.4, 1 mM EDTA, 2 mM EGTA, 150 mM NaCl, 1% NP40, 0.5% DOC, 0.1% SDS). Biotinylated surface proteins were precipitated with immobilized streptavidin beads and analyzed by WB with appropriate antibodies.

### Glutathione S-Transferase (GST) Pull-Down

Glutathione S-transferase (GST)-HIP1R_1–632_, GST-HIP1R_633–822_ and GST-HIP1R_823–1068_ motif were expressed in *E. coli* strain BL21 (DE3) and induced using 0.1 mM IPTG at 16°C overnight. Proteins were purified using GST Spin Purification kit (16106, Thermo Fisher Scientific, Waltham, MA, USA) in the presence of protease inhibitor cocktail tablets (04693116001, Roche Diagnostics, Indianapolis, IN, USA) according to manufacturer’s protocols. High density cultured neurons were lysed with RIPA buffer (Beyotime, Shanghai, China) and incubated with 2 μg of purified proteins overnight at 4°C. Forty microliter glutathione sepharose beads were added and the mixture was incubated for another 3 h at 4°C. After washing for five times, beads were resuspended in 40 μl sample buffer for western blot analysis.

### Immunoprecipitation

For IP, cultured hippocampal neurons with high density or hippocampus tissue from mouse brains were lysed with RIPA buffer and centrifuged at 12,000× *g* for 20 min. The supernatant was collected and the protein concentration was adjusted to 1 μg/μl. The corresponding antibody was added to 400 μl supernatant and incubated overnight at 4°C. Then 40 μl of protein A-sepharose beads was added to incubate for another 2 h. The sample was rinsed two times with binding buffer and three times with 1× PBS to remove non-specific interactions. After final centrifugation, the sample was incubated with sample buffer, boiled and used for western blot analysis.

### Western Blot Assay

Western blot assay was carried out as previously described (Wang et al., 2012; Lu et al., 2015). The proteins were separated by SDS-PAGE and transferred onto nitrate cellulose membranes. After blocking in 0.1% Tween-20 in Tris-buffered saline (TBST) containing 5% skimmed milk (for non-phosphorylated proteins) or 5% BSA (for phosphorylated proteins), the membranes were incubated with corresponding primary antibodies at 4°C overnight. After washing three times with TBST, the membranes were incubated with horseradish peroxidase-conjugated secondary antibodies for 1 h at room temperature, and detection performed with ECL (Pierce) system. Blots were analyzed using Quantity One software (Bio-Rad) and the original blots are provided (Supplementary Figure S1).

### Statistics

Statistical analyses were performed in GraphPad Prism 5 (GraphPad Software, La Jolla, CA, USA) using Student’s *t*-test (for sample pairs), one-way ANOVA (for three or more samples) followed with Tukey’s *post hoc* test, as indicated in figure legends. Results are shown as mean ± SEM, and n refers to the number of cells or experimental repeats which is specified individually in figure legends. All conditions statistically different from controls are indicated: *p < 0.05; **p < 0.01; ***p < 0.001; ****p < 0.0001. ns, not significant *p* > 0.05.

## Results

### HIP1R Knockdown at Early Stage Suppresses Neurite Initiation and Dendritic Branching

**Figure 1:**
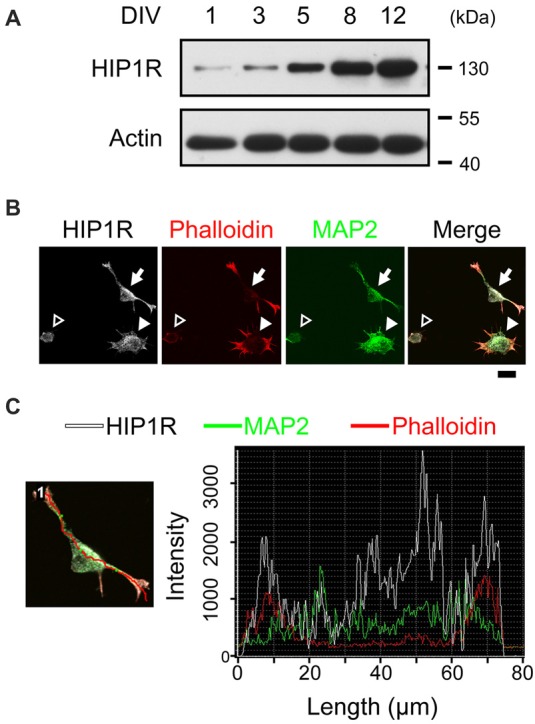
Huntington-interacting protein 1-related protein (HIP1R) co-localizes with actin during neurite initiation. **(A)** Expression of HIP1R in cultured hippocampal neurons at different time points. **(B)** Representative images of HIP1R expression in cultured hippocampal neurons. HIP1R (gray), phalloidin (red) and MAP2 (green). Open arrowhead, neurite initiation stage 1a; filled arrowhead, stage 1b; filled arrow, stage 2. Scale bar, 10 μm. **(C)** Representative analysis of fluorescence intensity distributions of HIP1R and F-actin in neuron. Line scan method was used to detect the intensities of fluorescent signals (right panel) along the red line in the example neuron (left panel).

Our previous study showed that HIP1R is increasingly expressed from the early embryo stage to the adult stage in the hippocampal tissue (Peng et al., 2017), and here we also examined HIP1R expression in cultured hippocampal neurons at different developmental stages. Immunoblots showed that HIP1R was expressed as early as DIV 1, with the expression level gradually increased from DIV 1 to DIV 12 (Figure 1A). We therefore asked whether HIP1R could also participate in earlier stage of neuronal development, such as neuritogenesis. As actin aggregations determine the site of neurite initiation (Dotti et al., 1988; Zhang et al., 2016), we first examined the co-location of actin and HIP1R at different stages of neuritogenesis. Consistent with previous result (Zhang et al., 2016), we could observe that neurons fixed 4–5 h after plating had thick and evenly distributed actin in stage 1a (Figure 1B, open arrowhead), one or two main actin aggregations in stage 1b (Figure 1B, filled arrowhead), and one or two short MAP2-containing neurites in stage 2 (Figure 1B, filled arrow). At this time, we found that HIP1R co-localized with MAP2 at the initiate segment of neurites and the cell body. More interestingly, HIP1R was enriched at the end of each extended neurite and co-localized with F-actin (Figures 1B,C), represented by pallolidin positive staining, indicating a possible role of HIP1R in neurite initiation.

**Figure 2:**
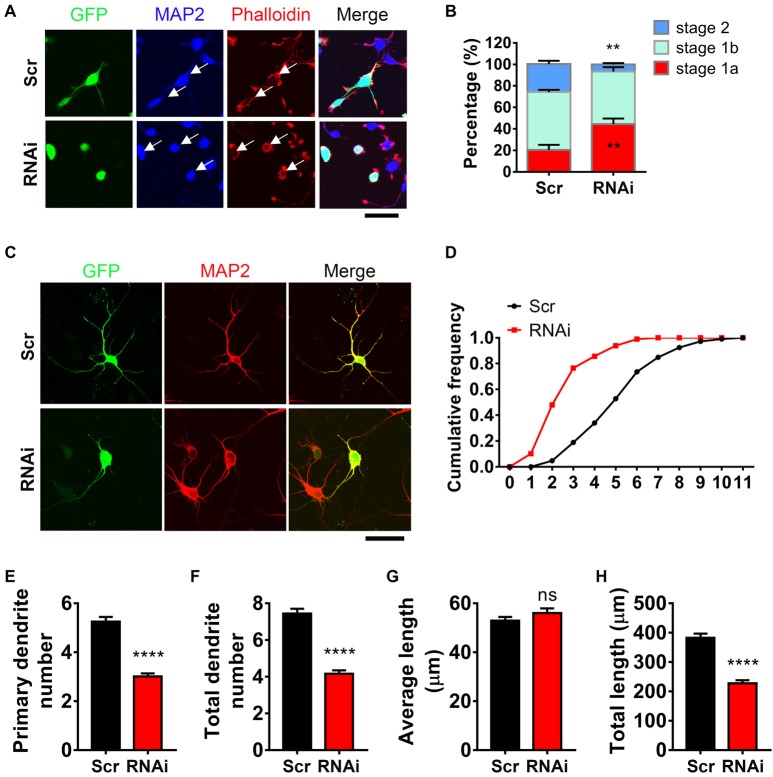
HIP1R is necessary for inducing neurite initiation and dendritic branching. **(A)** Representative images of cultured neurons electroporated with Scrambled-RNAi (Scrambled) or HIP1R-RNAi. GFP (green), MAP2 (blue) and phalloidin (red). Arrows indicate GFP-positive cells expressing Scrambled-RNAi or HIP1R-RNAi. Scale bar, 20 μm. **(B)** Quantification of neurite initiation (*n* = 3,749 neurons for the Scrambled group and 3,045 neurons for the RNAi group). **(C)** Representative images of day *in vitro* (DIV) 5–6 cultured neurons electroporated with Scrambled-RNAi or HIP1R-RNAi. MAP2 was used to outline the dendrites. Scale bar, 20 μm. **(D)** Cumulative distribution of primary dendritic number showed that HIP1R-RNAi neurons had fewer primary branches than Scrambled control. **(E–H)** Quantification of dendritic number and dendritic length in the Scrambled and HIP1R-RNAi groups (*n* = 106 and 151 neurons from more than three independent experiments). Mean ± SEM; **p < 0.01, ****p < 0.0001; ns, not significant; unpaired two-tailed *t*-test. Scr, scrambled-RNAi.

To examine whether HIP1R was necessary for neurite initiation, we transfected neurons with HIP1R specific RNAi via electroporation to knockdown HIP1R expression before plating. Neurite initiation was then detected 6 h after plating to view all three initiation stages. We found that HIP1R knockdown robustly damaged the formation of actin aggregates (Figure 2A). In addition, the population of neurons stayed in stage 1a were significantly larger than it was in control group, and the population in stage 2 were significantly less (Figure 2B), indicating a disordered neurite initiation with HIP1R deficiency.

Next, we examined the role of HIP1R in dendritic arborization at DIV 6 after 5 days of HIP1R knockdown. Transfected neurons were indicated by GFP signal and dendrites were outlined by MAP2 (Figure 2C). After HIP1R knockdown, the primary dendrite number was decreased compared to scrambled control, showed by cumulative frequency analysis (Figure 2D) and the average dendritic number count (Figure 2E). The number of total dendritic branches also showed a dramatically decrease in HIP1R deficient neurons (Figure 2F). Accordingly, the total length of dendrites was decreased with the average length unchanged (Figures 2G,H). Taken together, the results demonstrate that HIP1R plays a critical role in neurite initiation and dendritic arbor growth.

### HIP1R Is Necessary for EGF Induced Acceleration of Neuronal Morphogenesis

**Figure 3:**
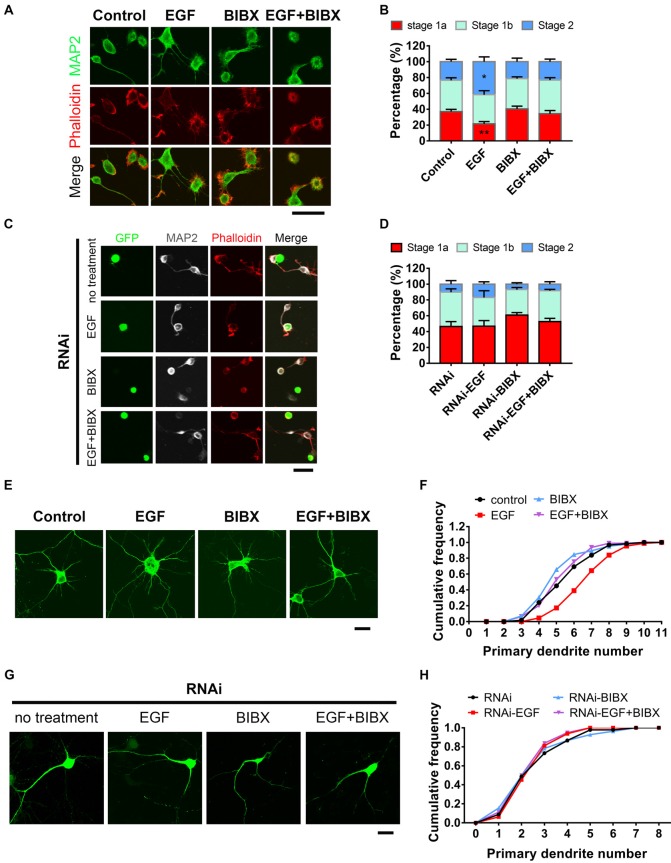
HIP1R is necessary for epidermal growth factor (EGF) induced-acceleration of neurite initiation and dendrite arbor growth. **(A)** Representative images of DIV 0 neurons treated with EGF (50 ng/ml), BIBX (0.5 μM) or EGF+BIBX and labeled for MAP2 (green), pallolidin (red), harvested 4–5 h after plating. Scale bar, 20 μm. **(B)** Quantification of neurite initiation after EGF treatment (*n* = 4,100 neurons for Control, 4,372 neurons for EGF, 5,003 neurons for BIBX and 3,913 neurons for EGF+BIBX treated neurons; ≥3 independent cultures; unpaired two-tailed *t*-test). **(C,D)** Representative images **(C)** and neurite initiation stage quantification **(D)** of HIP1R-RNAi neurons EGF, BIBX or EGF+BIBX, harvested 5–6 h after plating (*n* = 1,419 neurons for RNAi, 1,040 neurons for EGF, 1,642 neurons for BIBX and 1,368 neurons for EGF+ BIBX treated neurons; three independent cultures; unpaired two-tailed *t*-test). Scale bar, 20 μm. **(E)** Representative images of DIV 6 neurons treated with EGF, BIBX or EGF+BIBX. Scale bar, 20 μm. **(F)** Cumulative distribution of primary dendritic numbers of control, EGF, BIBX or EGF+BIBX treated groups (*n* = 62, 87, 85 and 94, respectively; three independent cultures). **(G)** Representative images of HIP1R-RNAi neurons without treatment and treated with EGF, BIBX or EGF+BIBX at DIV 6. Scale bar, 20 μm. **(H)** Cumulative distribution of primary dendritic numbers of HIP1R-RNAi neurons with or without treatment (*n* = 64, 64, 83 and 80, respectively; three independent cultures). All data are presented as mean ± SEM; *p < 0.05; **p < 0.01.

Previous work showed that EGFR activation contributes to neurite outgrowth in PC12 cells and cortical neurons (Goldshmit et al., 2004a,b). To gain insight into the role of EGFR at early developmental stages in hippocampal neurons, we applied EGF (50 ng/ml), a specific ligand for EGFR, into the medium to activate EGFR at the time of neuron plating for 5–6 h. Result showed that EGF was capable of promoting actin aggregation and neurite initiation (Figure 3A), which was consistent with the previous work (Goldshmit et al., 2004a). EGF stimulated neurons revealed an accelerated neuronal development, which was shown by a shifting neuronal population from early to later stage (Figure 3B). To rule out the possibility that this phenomenon was elicited by receptors other than EGFR (Tsai et al., 2010; Puehringer et al., 2013), we treated the neurons with BIBX-1382, an EGFR specific inhibitor, alone or in combination with EGF. BIBX alone showed no effect on neurite initiation. However, when combined with EGF, BIBX effectively blocked the neuritogenic role of EGF (Figure 3B). These results together indicate that EGFR activation is required for actin aggregation and neurite initiation.

To further investigate EGFR’s role in dendrite development, we applied EGF to neurons throughout the whole culturing process until DIV 5–6 for analysis. MAP2 staining clearly outlined the dendritic arbors and through which we found that EGF increased the number of primary dendrite (Figures 3E,F) without affecting dendritic average length (Supplementary Figure S2C). BIBX completely blocked the increase of primary dendritic branches induced by EGF and even caused decreases of dendritic branching and dendrite length without EGF stimulation (Supplementary Figures S2A–D), demonstrating a specific role of the activation of EGFR in dendritic branching.

To examine whether HIP1R is involved in EGF induced neurite initiation and dendritic outgrowth, we knocked down HIP1R expression by RNAi before EGF stimulation. In that case, EGF could promote neither neurite initiation (Figures 3C,D) nor dendritic arborization (Figures 3G,H) in the absence of HIP1R, suggesting the acceleration of neuronal development induced by EGF stimulation was abolished by HIP1R knockdown. In addition, we found that the application of BIBX leaded to a slight decrease in the occurrence of protrusions in RNAi neurons, but did not lead to a statistically significance (Figures 3C,D). RNAi treatment abolished EGF stimulated primary dendrite number and total length increase, with or without BIBX treatment (Supplementary Figures S2E,H), indicating a lineage involvement of BIBX and HIP1R in EGFR mediated primary dendrite initiation and dendrite extension. However, BIBX treatment could further reduce the RNAi-induced reduction in overall dendritic branching (Supplementary Figure S2F) and increase the average length of dendrite (Supplementary Figure S2G). It is possible that the RNAi knockdown efficiency was not high enough to elicit significant functional consequence in overall dendritic branching or the phosphorylated EGFR could be endocytosed in more pathways other than HIP1R. All these results demonstrated that HIP1R is involved in the EGF-induced enhancement of neuronal development.

Since BDNF has been demonstrated to be one of the most well-studied extrinsic neurotrophins for dendrite development (Dijkhuizen and Ghosh, 2005; Alder et al., 2012; Lazo et al., 2013), we also examined its effect on neuronal development. We found that BDNF exerts no effect on neurite initiation, but promotes neurite extension (Supplementary Figures S3A–F), demonstrating that BDNF-TrkB activity has a selective effect in the early stage of neuronal development. Moreover, we found that neither the TrkB expression level nor its surface expression level (Supplementary Figures S3G–J) was changed after HIP1R knockdown, indicating that HIP1R is not involved in TrkB expression. To further explore the relationship between these two proteins, we detected the localization of TrkB and HIP1R in HeLa cells. The result showed that TrkB and HIP1R are differently distributed even after BDNF stimulation (Supplementary Figure S3K). In contrast, EGFR obviously co-localized with HIP1R after EGF stimulation (Supplementary Figure S3L). These results indicated that HIP1R is selectively involved in EGFR, but not TrkB internalization.

### HIP1R Mediates Ligand-Induced Endocytosis of EGFR and Activation of MAP Kinase (ERK) and Akt Signaling Pathways

**Figure 4:**
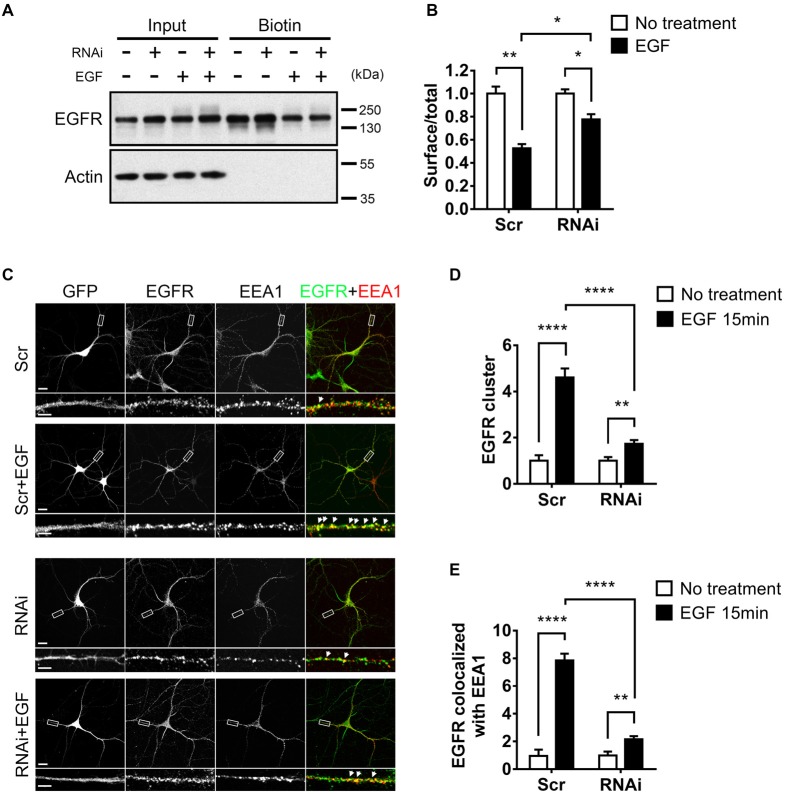
HIP1R knockdown affects the internalization of EGF receptor (EGFR). **(A)** Total and surface EGFR expression after EGF stimulation in cultured hippocampal neuron at DIV 12. **(B)** Quantification of EGFR expression. **(C)** Representative images of neurons, with or without EGF stimulation. GFP (gray), EGFR (green) and early endosomal autoantigen 1 (EEA1; red). GFP indicates successful transfection of Scrambled-RNAi or HIP1R-RNAi. Filled white arrows showed colocalized EGFR and EEA1 clusters. Scale bar, 20 μm for whole cell and 10 μm for partial enlarged detail. **(D,E)** Quantification of EEA1-colocalized EGFR clusters (*n* = 22 neurons for Scrambled, 23 neurons for Scrambled+EGF, 25 neurons for RNAi, and 25 neurons for RNAi+EGF groups). Mean ± SEM; *p < 0.05; **p < 0.01; ****p < 0.0001; three independent experiments; unpaired two-tailed *t*-test.

As EGF activates EGFR for neuronal development and HIP1R was found to be critical for EGF-induced events, we next asked whether the activity of EGFR was mediated by HIP1R. Having been reported that the overexpression of HIP1R prevents the ligand-induced endocytosis and degradation of EGFR in NIH3T3 fibroblasts (Hyun et al., 2004b), we evaluated the effect of HIP1R on EGFR stability after EGF stimulation in neurons by RNAi strategy. Surface biotinylation assay was applied in neurons with or without HIP1R knockdown to determine the expression level of membrane-associated EGFR. Strikingly, HIP1R-RNAi led to a stronger expression of surface EGFR (Figure 4A), as well as of the total EGFR (Figures 4A, 6A,B) in neurons, compared to scrambled control. EGF stimulation caused a percentage decrease of surface expressed EGFR in scrambled control group (47.36 ± 2.20%), and the decrement was much less when HIP1R expression was knocked down (22.43 ± 2.55%; Figure 4B), suggesting that the endocytosis of EGFR could be affected after HIP1R knockdown.

**Figure 5:**
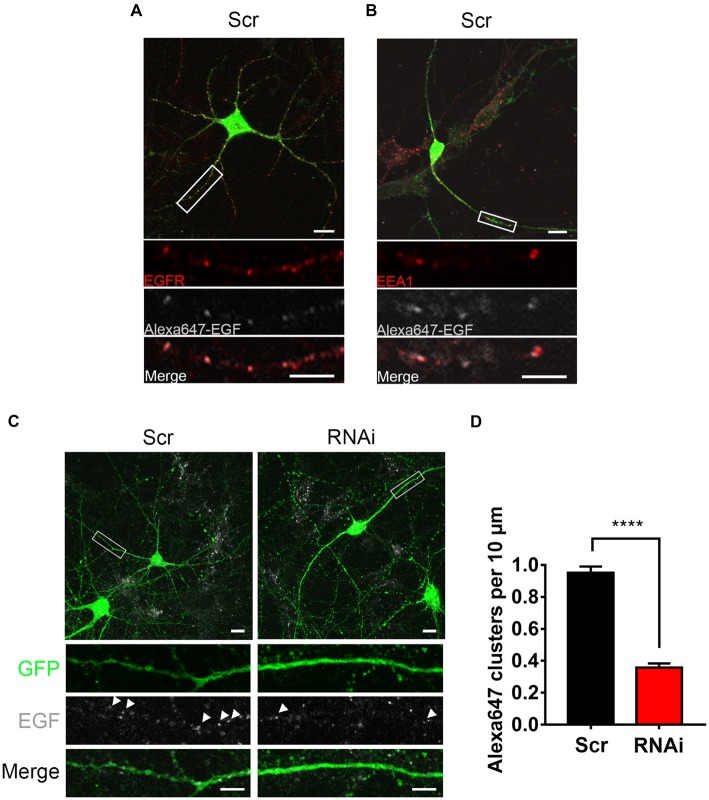
HIP1R knockdown affects the internalization of EGF. **(A)** Representative images of control neurons after a 20 min-treatment of Alexa^647^-EGF stained against EGFR (red). GFP (green) and Alexa^647^-EGF (gray). Scale bar, 20 μm. **(B)** Representative images of Alexa^647^-EGF treated control neurons stained against EEA1 (red). GFP (green) and Alexa^647^-EGF (gray). Scale bar, 20 μm. **(C)** Representative images of Alexa^647^-EGF treated control and knockdown neurons. Scale bar, 20 μm. **(D)** Quantification of EGF clusters (*n* = 73 neurons for Scrambled and 51 neurons for RNAi). Mean ± SEM; ****p < 0.0001; three independent experiments; unpaired two-tailed *t*-test.

**Figure 6:**
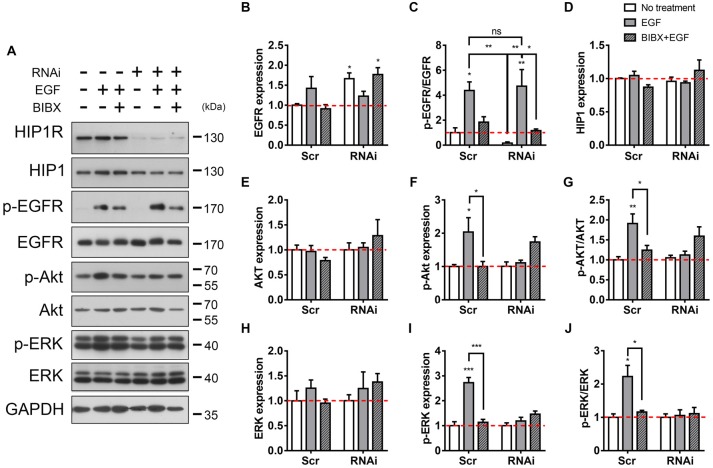
HIP1R knockdown attenuates EGFR downstream signaling. **(A)** The detection of EGFR and downstream Akt and ERK activity after EGF stimulation. **(B)** Quantitative analysis of EGFR expression after EGF stimulation in Scrambled and HIP1R-RNAi group. **(C)** Quantitative analysis of phosphorylated EGFR after EGF stimulation. **(D)** Quantitative analysis of HIP1. **(E–G)** Quantification of Akt **(E)**, p-Akt **(F)** and the ratio of p-Akt/Akt **(G)** level. **(H–J)** Quantification of ERK **(H)** p-ERK **(I)** and the ratio of p-ERK/ERK **(J)** level. Normalized by GAPDH and compared with untreated control. Mean ± SEM; ns, not significant; *p < 0.05; **p < 0.01; ***p < 0.001; *n* = 4 independent experiments; unpaired two-tailed *t*-test.

To better dissect the endocytic portion of EGFR, we further analyzed the EGFR clusters colocalized with EEA1, an essential component of the endosomal fusion machinery (Simonsen et al., 1998; Ramanathan and Ye, 2012). We could see that compared to the small and diffuse puncta in non-stimulated condition, after EGF stimulation, EGFR puncta formed bigger and brighter clusters which mostly colocalized with EEA1 in both neuron and HeLa cell, and the puncta number was increased (Figure 4C, Supplementary Figure S3L), showing that EGFR was internalized into early endosomes. However, in RNAi group, a large population of EGFR still existed as small puncta, and were not co-localized with EEA1 even after EGF stimulation (Figure 4C). Quantitative data showed that the EGF-induced increase of EGFR clusters was largely abolished in RNAi group (Figures 4C,D). For the co-localization of EGFR and EEA1, although EEA1-colocalized EGFR clusters were increased after ligand stimulation, such increment was also largely abolished in HIP1R knockdown group (Figures 4C,E), indicating that HIP1R mediates the internalization of EGFR after EGF stimulation.

To investigate HIP1R’s role in EGFR internalization more directly, we examined the internalized Alexa^647^ labeled EGFR ligand EGF (Alexa^647^-EGF) to visualize the internalization of EGF-EGFR complex. Alexa^647^-EGF was incubated with neurons to allow for internalization. The remaining surface-bound EGF was then striped with acid buffer. Distinguishable Alexa^647^-EGF puncta inside the cytoplasm was detected, which were co-localized with EGFR or EEA1 (Figures 5A,B), indicating an internalization of EGF-EGFR complex in the endosomes. However, the internalized EGF clusters were much less in RNAi neurons compared to control neurons (Figures 5C,D), showing a suppression of EGF internalization after HIP1R knockdown. Taken together, these results demonstrate that HIP1R is necessary for the ligand induced early endocytosis of EGFR.

It is known that the binding of EGF to the surface EGFR triggers the dimerization and autophosphorylation of EGFR, and then induces the cellular internalization of EGFR (Iwashita and Kobayashi, 1992; Heldin, 1995; Lemmon and Schlessinger, 2010). We then examined whether HIP1R knockdown also affected the phosphorylation of EGFR. Immunoblots showed level of the EGF-induced EGFR phosphorylation in RNAi group was as high as that in the scrambled group, both of which could be readily block by BIBX (Figures 6A–C). Together with the previous result, this result indicates that though HIP1R mediates the endocytosis of EGFR, it does not affect the EGF-stimulated EGFR phosphorylation which is prior to the endocytosis.

Next, we examined EGFR-activated downstream signal transduction pathways involved in neurite outgrowth. Akt plays a role in promoting neurite outgrowth (Niyomchan et al., 2015; Nishimoto et al., 2016), which makes it a potential candidate. Immunoblotting result showed that in scrambled control, EGF treatment increased the phosphorylation level of Akt, which could be blocked by BIBX (Figures 6A,E–G). However, alterations in neither the expression nor the phosphorylation of Akt was found in RNAi neurons and BIBX did not further inhibit the phosphorylation of Akt (Figures 6A,F,G), suggesting that HIP1R knockdown abolished EGF-induced Akt activation. ERK was another EGFR downstream protein which plays a critical role in cell development, differentiation and survival, and also participates in the neurite outgrowth (Xiang et al., 2006; Fu et al., 2011; Lu et al., 2014). The same like Akt, in scrambled group, EGF-induced ERK phosphorylation was increased without the change in protein expression level (Figures 6A,H–J). However, HIP1R knockdown suppressed the enhancement of ERK phosphorylation and the p-ERK/ERK ratio induced by EGF, with or without BIBX (Figures 6A,I,J). Taken together, these results indicated that HIP1R regulates the activation of EGFR downstream proteins Akt and ERK.

Additionally, the expression level of the HIP1R homolog HIP1 was found to have no change with or without stimulation in both control and HIP1R knockdown groups (Figures 6A,D), demonstrating that there is no unspecific targeting on HIP1 by HIP1R RNAi.

### HIP1R Interacts With EGFR by Its Amino Acids 633–822 Fragment Which Is Critical for EGF-Induced EGFR Endocytosis

**Figure 7:**
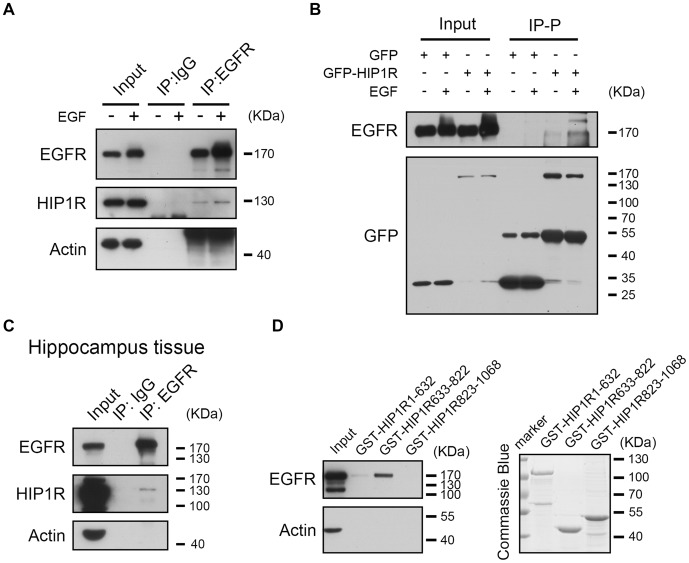
HIP1R interacts with EGFR via amino acid 633–822 fragment. **(A,B)** EGFR interacts with HIP1R in cultured hippocampal neurons harvested at DIV 6–8. **(A)** Neuron lysates were immunoprecipitated with an anti-EGFR antibody. Lysates (input) and precipitates (Immunoprecipitation, IP: IgG and IP: EGFR) were blotted with anti-EGFR, anti-HIP1R and anti-actin antibodies. **(B)** Neurons electroporated with GFP-HIP1R or GFP-control were immunoprecipitated with anti-GFP antibody. **(C)** EGFR interacts with HIP1R in hippocampus tissue dissected from 6-week-old male mice. **(D)** GST-pull down of EGFR using purified GST-HIP1R_1–632_, GST-HIP1R_633–822_ and GST-HIP1R_823–1068_ in cultured hippocampal neurons harvested at DIV 6–8.

Previous work has shown that HIP1R can interact with EGFR by co-IP assay (Co-IP) in HEK293T cells (Bradley et al., 2007). To verify the interaction in neurons, we also used the Co-IP assay and found that HIP1R was detectable in the immunoprecipitates of endogenous EGFR in cultured hippocampal neurons (Figure 7A). In addition, an increase of HIP1R in the IP fraction was also detected after EGF application, indicating an activation-dependent enhancement of the interaction between HIP1R and EGFR (Figure 7A). As the HIP1R antibody was not suitable for endogenous IP, we overexpressed myc-HIP1R and immunoprecipitated the HIP1R with an anti-myc antibody. EGFR was also detected in the IP pellet and the IP level was elevated by EGF treatment (Figure 7B). Though it was more difficult to perform Co-IP assay in brain tissue, we were still able to detect a light signal of HIP1R in the IP pellet of EGFR (Figure 7C). Moreover, amino acids fragment 633–822 of human HIP1R (HIP1R_633–822_) was suggested to be responsible for the interaction between HIP1R and EGFR in HEK293T cells (Bradley et al., 2007). To verify the interaction domain, we purified GST fused-HIP1R_1–632_, HIP1R_633–822_ and HIP1R_823–1068_ proteins and performed GST pull-down assay in brain tissue lysate but failed to detect any positive interaction (data not shown). However, when we used cultured hippocampal neurons to perform the GST pull-down assay, we could find that EGFR vividly bound to HIP1R_633–822_ fragment (Figure 7D). This result indicates that amino acids 633–822 sequence mediates the interaction between HIP1R and EGFR.

**Figure 8:**
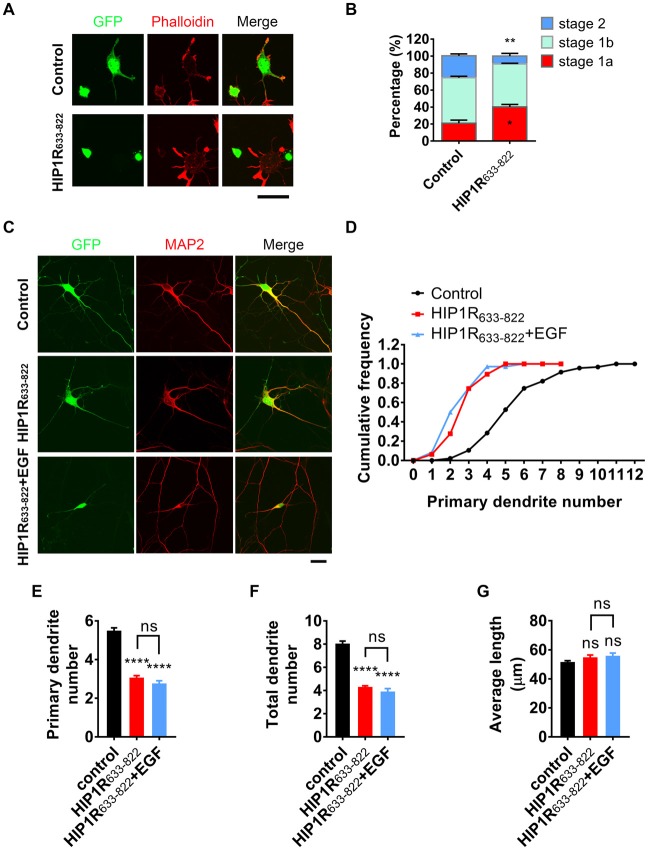
HIP1R_633–822_ overexpression disturbs neurite initiation and dendritic branching. **(A)** Representative images of cultured neurons electroporated with GFP-control) and GFP-HIP1R_633–822_ constructs, fixed 5–6 h after plating, and labeled for GFP (green), MAP2 (blue) and phalloidin (red). **(B)** Quantification of neurite initiation in control and HIP1R_633–822_ groups (*n* = 1,150 and 1,368 neurons). **(C)** Representative images of cultured neurons transfected with GFP or GFP-HIP1R_633–822_ and fixed at DIV 5–6. MAP2 staining outlined the dendrites. Scale bar, 20 μm. **(D)** Cumulative distribution of primary dendritic number of control and HIP1R_633–822_ groups. **(E–G)** Quantification of dendritic number and dendritic length of neurons in control and HIP1R_633–822_ groups (*n* = 86 and 47 neurons). Data are presented as mean ± SEM, ns, not significant > 0.05; *p < 0.05; **p < 0.01; ****p < 0.0001; ≥3 independent experiments; unpaired two-tailed *t*-test.

We wondered if HIP1R_633–822_ participated in HIP1R and EGFR-mediated neuronal development. Therefore, we overexpressed HIP1R_633–822_ fragment in cultured neurons to examine if it exerted any dominant negative effect. Similar to HIP1R deficient neurons, actin aggregation and neurite initiation were delayed when overexpressing HIP1R_633–822_ in cultured hippocampal neurons (Figures 8A,B), suggesting that HIP1R_633–822_ abolished the function of endogenous HIP1R, leading to the impairment of neuritogenesis. In addition, overexpression of HIP1R_633–822_ also abolished the enhancement of EGF-induced dendritic branching (Figures 8C–G). All these results indicate that HIP1R_633–822_ suppressed neurite initiation and dendritic arbor growth by disturbing the interaction of HIP1R and EGFR.

**Figure 9:**
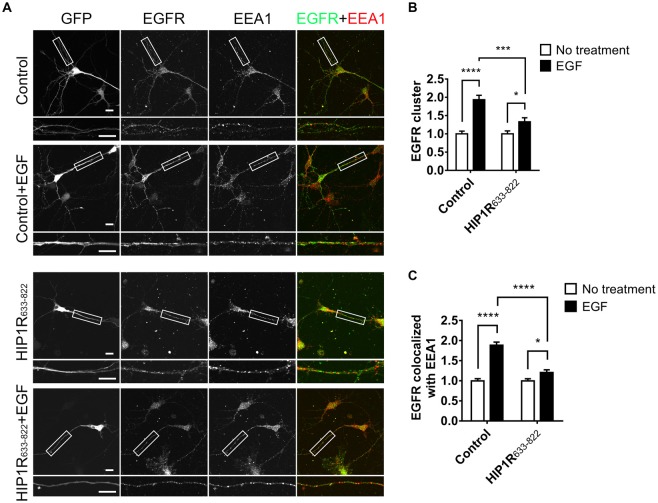
HIP1R_633–822_ overexpression influences the internalization of EGFR. **(A)** Representative images of neurons electroporated with GFP or GFP-HIP1R_633–822_ constructs and treated with EGF for 15 min, labeled for GFP, EGFR and EEA1. Scale bar, 20 μm. **(B,C)** Quantification of EGFR clusters **(B)** and the colocalization of EGFR and EEA1 clusters **(C)** in control and HIP1R_633–822_ groups (*n* = 34 and 59 neurons for control groups, and 42 and 41 neurons for HIP1R_633–822_ groups). Mean ± SEM, *p < 0.05; ***p < 0.001; ****p < 0.0001; *n* = 3 independent cultures; unpaired two-tailed *t*-test.

To further investigate the dominant negative effect of HIP1R_633–822_ overexpression, we examined the endocytosis of EGFR and the activation of downstream signals. Result showed that EGF-induced EGFR cluster increase was largely abolished in HIP1R_633–822_ group (Figures 9A,B). Moreover, elevated colocalization of EGFR with EEA1 after EGF treatment was also abolished in HIP1R_633–822_ group (Figure 9C). Taken together, these results showed that EGFR endocytosis was abolished after HIP1R_633–822_ expression.

**Figure 10:**
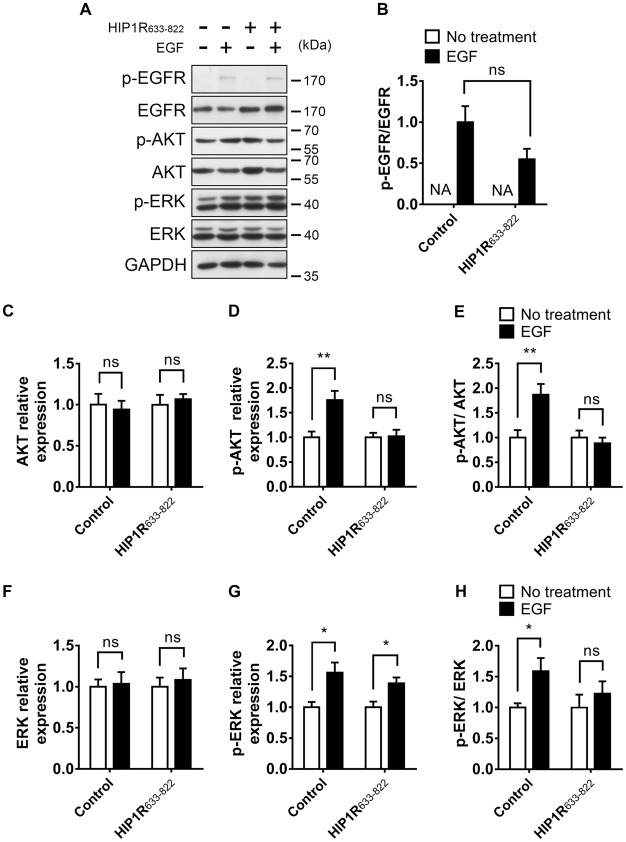
Overexpression of HIP1R_633–822_ attenuates EGFR downstream signaling. **(A)** EGFR, Akt and ERK expression and phosphorylation with or without EGF treatment, in DIV 12 neurons. **(B)** Quantitative analysis of phosphorylated EGFR level. NA, not applicable. **(C–E)** Quantification of Akt **(C)**, p-Akt **(D)** level and the p-Akt/Akt ratio **(E)**. Normalized by GAPDH and compared with untreated control. **(F–H)** Quantification of ERK **(F)**, p-ERK **(G)** level and the p-ERK/ERK ratio **(H)**. Normalized by GAPDH and compared with untreated control. Mean ± SEM; ns, not significant; *p < 0.05; **p < 0.01; *n* = 3 independent experiments; unpaired two-tailed *t*-test.

Second, we detected the activation of EGFR autophosphorylation and downstream signaling after HIP1R_633–822_ overexpression. Consistently, Similar as HIP1R knockdown, the EGFR expression level was increased in HIP1R_633–822_ group (Figure 10A). HIP1R_633–822_ did not affect the EGF-induced EGFR phosphorylation (Figures 10A,B), but abolished EGF-induced upregulation of Akt and ERK phosphorylation (Figures 10C–E for Akt and Figures 10F–H for ERK). Thus, the interaction of HIP1R and EGFR was necessary for the EGF-induced activation of PI3K-Akt and MAPK/ERK signaling pathway.

## Discussion

In summary, we confirmed that EGFR played a critical role in the patterning of neuronal morphology during early-stage development and found that HIP1R modulates the ligand-dependent endocytosis of EGFR, thereafter mediates the activation of downstream signaling pathways and regulates the development of neurite initiation and dendritic outgrowth.

EGF and EGFR are considered to be involved in the proliferation of the neuronal precursors (Kuhn et al., 1997; O’Keeffe et al., 2009; Fujimoto et al., 2016), but they also play more roles in neurons, such as promoting the neurite outgrowth (Goldshmit et al., 2004a,b; Tsai et al., 2010), and involving in the synaptic transmission and synaptic plasticity in cultured hippocampal neurons or rat hippocampus (Abe and Saito, 1992; Yamada et al., 1997). Here we show that EGFR is also involved in neurite initiation and dendritic branching in early stage development upon EGF stimulation. Such an effect could be achieved through both Akt and ERK pathway. Since activated Akt phosphorylates suppresses Raf kinase activity and the subsequent ERK activity via phosphorylation of Raf-1 (Moelling et al., 2002; Hatakeyama et al., 2003). Cross talk between Akt and ERK signaling has also been observed in HeLa cells (Gross and Rotwein, 2017), suggesting that upregulated Akt and downregulated ERK signaling could be both involved after EGFR activation in neurons. However, what we found in cultured hippocampal neurons after EGF stimulation was the boosted phosphorylation of both Akt and ERK, indicating that EGF could possibly elicited a synergistic effect of both pathways regarding to neurite development. Actually, Akt and ERK have both been demonstrated to be required for dendritic morphogenesis (Jaworski et al., 2005; Kumar et al., 2005).

Our previous study showed that RNAi mediated HIP1R deficiency dramatically suppresses dendritic growth and spine formation (Peng et al., 2017). However, the RNAi transfection or RNAi lentiviral infection were performed at DIV 6, when endogenous HIP1R had already existed and might have played some role in neuronal development much earlier. In this study, we delivered the RNAi into neurons before plating by electroporation assay. Compared to our previous results, we found a higher level of decrease in dendritic branching after HIP1R knockdown. In addition, just 6 h after HIP1R knockdown, we were able to find a delayed neurite initiation. Considering that there were undegraded HIP1R proteins remaining, the effect of HIP1R in neurite initiation could be very much underestimated. However, HIP1R knockout mice are viable and fertile and showed no obvious morphological disorders (Hyun et al., 2004a). This discrepancy might be due to the compensatory role of HIP1 in HIP1R function, as overexpression of HIP1 rescues the degenerative phenotypes in HIP1R/HIP1 double-knockout mice (Hyun et al., 2004a).

In addition, HIP1R has been suggested to bearing endocytic function in heterologous cells, and yet no further evidence was given in the CNS. We found in our study that HIP1R was able to interact with EGFR in neurons *in vivo* and participated in EGFR endocytosis in neurons. Moreover, HIP1R was much more readily recruited to EGFR complex after EGF treatment, demonstrating the interaction of these two proteins is ligand-binding-dependent. However, whether it depends on EGFR-mediated phosphorylation of HIP1R (Ames et al., 2013) needs to be further proven. We also detected an increased expression level of EGFR after HIP1R knockdown. It might belong to an accumulation of endocytic complex after the loss of HIP1R (Engqvist-Goldstein et al., 2004), since we tested the degeneration of EGFR 1.5 h after EGF treatment and found a prolonged half-life of EGFR (data not shown). In addition, we have confirmed that amino acids 633–822 of HIP1R mediated its interaction with EGFR in neurons. Overexpression of HIP1R_633–822_ had a dominant negative role that impairs the neuronal morphogenesis and EGFR internalization, further supporting our finding that HIP1R-dependent EGFR endocytosis is necessary for neurite initiation and dendritic arborization. However, when used HIP1R_1–632_ as a control, we have found a weak signal of EGFR in the pull-down pellet. It might be due to that the 302–348 a.a. fragment of HIP1R was responsible for HIP1R dimerization (Legendre-Guillemin et al., 2002) and the HIP1R_1–632_ fragment reserved the capability of binding to the endogenous full length HIP1R. Therefore, still a small amount of EGFR could be pulled down by HIP1R_1–632_ via its interaction with endogenous EGFR. Moreover, HIP1R is reported to interact directly with another multidomain adaptor protein, CIN85 (Kowanetz et al., 2004), whose interaction with Cbl is necessary for the Cbl-mediated down-regulation of EGFR in non-neuronal cells (Soubeyran et al., 2002). Therefore, it is possible that the interaction between CIN85 and HIP1R is also necessary in ligand-dependent endocytosis of EGFR.

Previous work showed that knocking down of HIP1R in HeLa cells led to a reduction of receptor mediated transferrin uptake, and an accumulation of actin and endocytic proteins near cell cortex (Engqvist-Goldstein et al., 2004). We also detected an accumulation of actin in the cell body of cultured neuron after HIP1R knocking down (data not shown). This could be possibly due to the eliminated interaction between cortactin and HIP1R, as cortactin promotes the formation as well as stabilizes the branched actin networks (Weed et al., 2000; Weaver et al., 2001), and interacts with HIP1R (Le Clainche et al., 2007). Our previous work has confirmed that HIP1R-Cortactin interaction is critical for dendrite development and spine formation (Peng et al., 2017), and we could suppose that this interaction may also play a role in neurite initiation and early dendritic branching.

## Author Contributions

QY, JX and JL designed the study. QY performed most of the experiments. LP performed the WB and ICC of EGFR expression. YW performed the GST-HIP1R_633–822_ purification and GST-pull down. QY, YL and LW analyzed the data. QY, JX and JL wrote and revised the article. All authors commented on the manuscript.

## Conflict of Interest Statement

The authors declare that the research was conducted in the absence of any commercial or financial relationships that could be construed as a potential conflict of interest.
