# Supplementary material for: Endocytic Adaptor Protein HIP1R Controls Intracellular Trafficking of Epidermal Growth Factor Receptor in Neuronal Dendritic Development
Source: Front Mol Neurosci. 2018 Dec 6;11:447. doi: 10.3389/fnmol.2018.00447 (PMC6291753; doi:10.3389/fnmol.2018.00447)
Supplement: Supplementary file 1 [file Data_Sheet_1.pdf]

Supplementary Material

Supplementary Figure Legends

Figure S1 All raw blots show both molecular weights and the blot insets for each main figure.

Figure S2 (Supplement to Figure 3) **(A-D)** Quantification of dendritic number and dendritic length of DIV 6 cultured neurons without treatment, and with EGF, BIBX or EGF-BIBX treatment. n = 62, 87, 85 and 94, respectively; 3 independent cultures; one-way ANOVA *Tukey's post hoc test*. **(E-H)** Quantification of dendritic number and dendritic length of DIV6 HIP1R knockdown neurons without treatment, and with EGF, BIBX or EGF-BIBX treatment. n = 64, 64, 83 and 80, respectively; 3 independent cultures; one-way ANOVA *Tukey's post hoc test*. All data are presented as mean ± SEM; *$p < 0.05$; **$p < 0.01$; ***$p < 0.001$.

Figure S3 The effects of BDNF on neurite initiation and dendrite arbor growth. **(A)** Representative images of DIV 0 neurons treated with or without BDNF (25 ng/ml) and labeled for MAP2 (green), pallolidin (red), harvested 4-5 hr after plating. Scale bar, 20 μm. **(B)** Quantification of neurite initiation after BDNF treatment (n = 2505 neurons for Control, 3245 neurons for BDNF treated neurons; ≥ 3 independent cultures; unpaired two-tailed *t-test*). **(C)** Representative images of DIV 6 neurons treated with or without BDNF. Scale bar, 20 μm. **(D-F)** Quantification of primary and total dendritic numbers, and dendritic average length of control and BDNF treated

groups. n = 125 neurons for Control, 105 neurons for BDNF treated group; ≥ 3 independent cultures; unpaired two-tailed *t-test.* Data are presented as mean ± SEM; ****$p < 0.0001$. **(G)** Representative images of surface (red) and total (green) TrkB distribution in control and HIP1R knockdown neurons. Scale bar, 20 μm. **(H-J)** Quantification of the total, surface and average (surface/total) intensity of TrkB in control and RNAi group. n = 106 and 125, respectively; 3 independent cultures; unpaired two-tailed *t-test.* Data are presented as mean ± SEM; ns, not significant. **(K)** Representative images of Hela cells transfected with GFP-HIP1R (green) and RFP-TrkB (red) with or without BDNF treatment. Scale bar, 10 μm. **(L)** Representative images of Hela cells transfected with GFP-HIP1R (green) with or without EGF treatment, stained by EGFR (red). Scale bar, 10 μm.
