# Supplementary figures and images for: Endocytic Adaptor Protein HIP1R Controls Intracellular Trafficking of Epidermal Growth Factor Receptor in Neuronal Dendritic Development
Source: Front Mol Neurosci. 2018 Dec 6;11:447. doi: 10.3389/fnmol.2018.00447 (PMC6291753; doi:10.3389/fnmol.2018.00447)

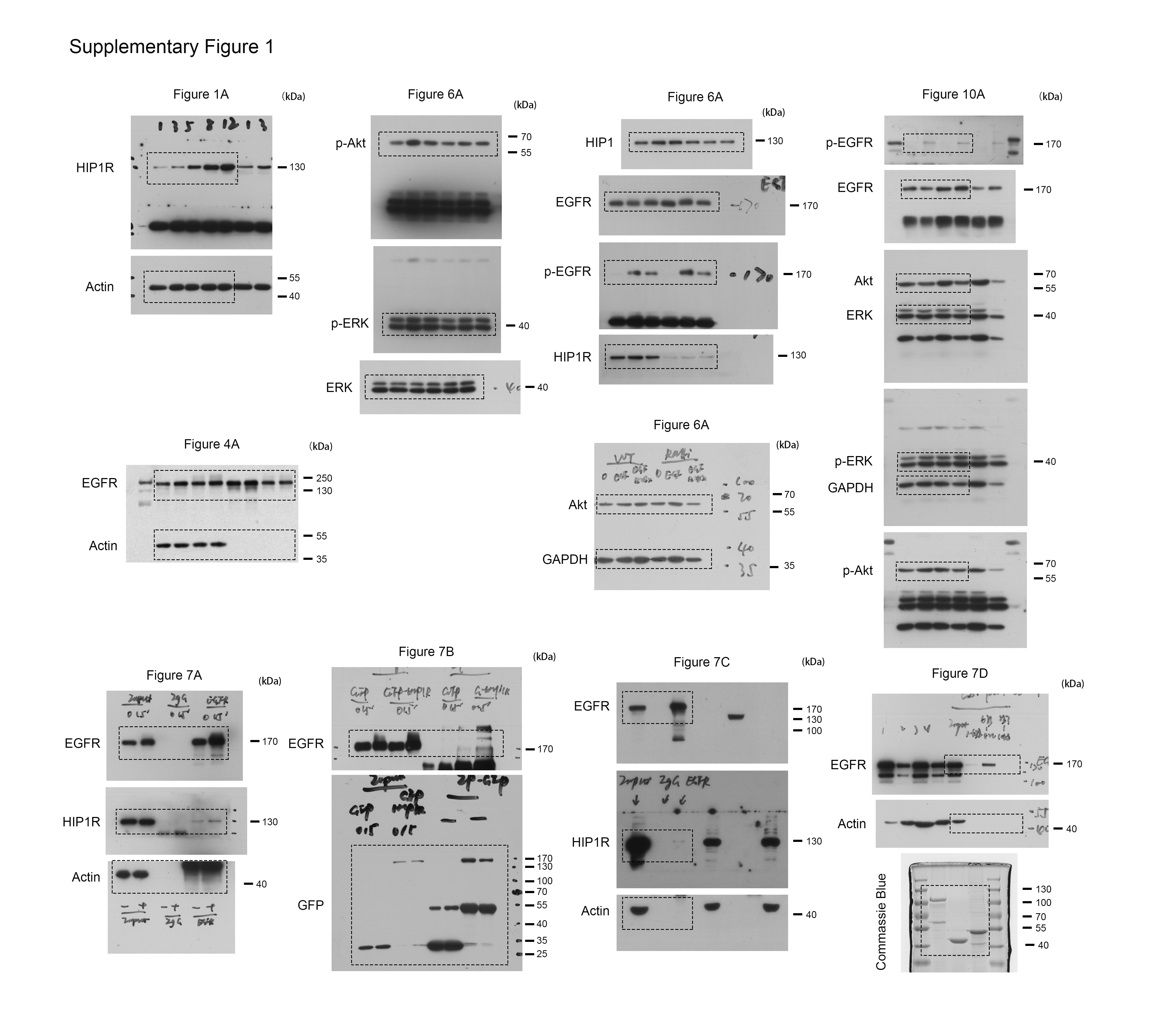

Supplement: Supplementary file 2 [file Image_1.tif]

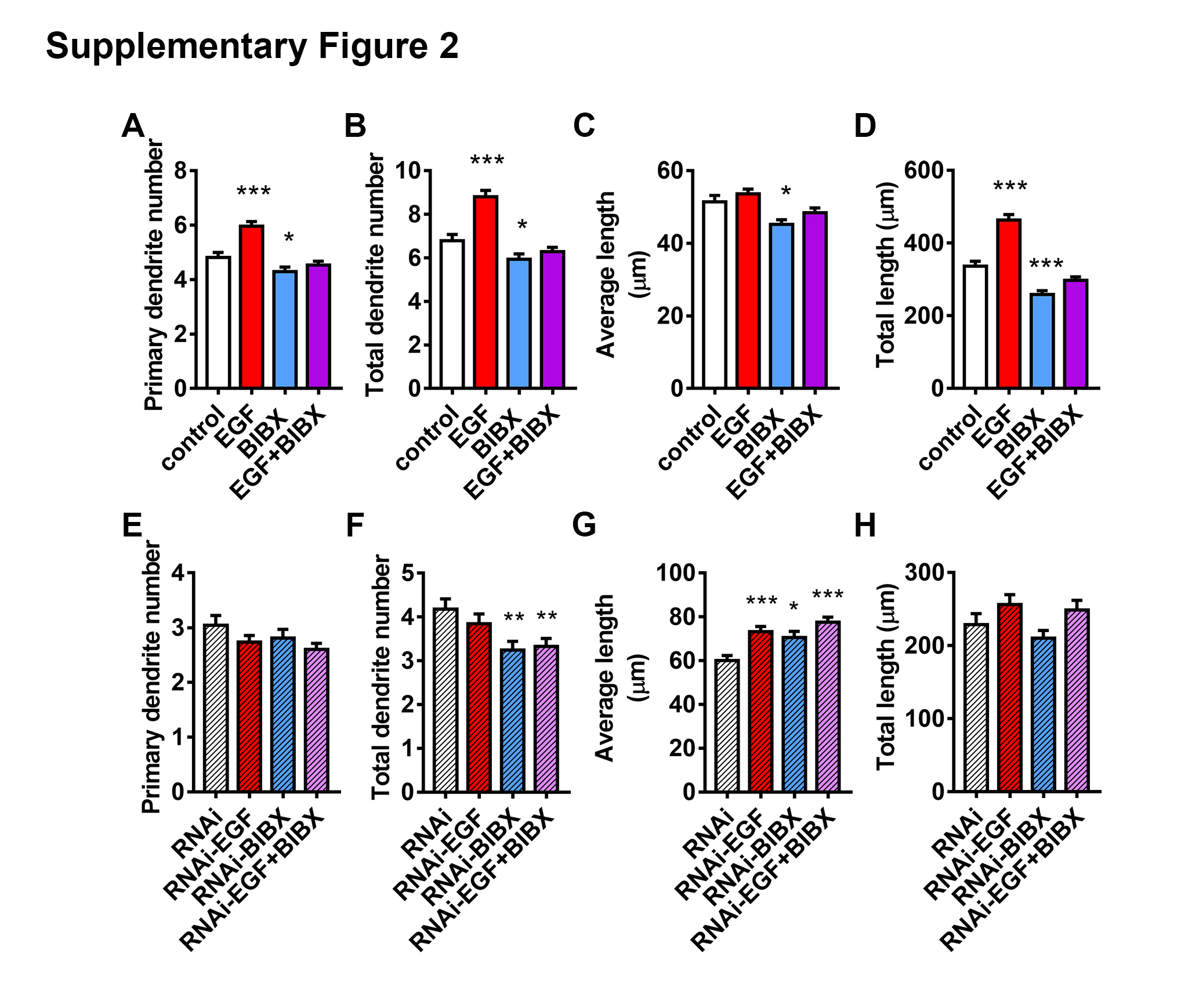

Supplement: Supplementary file 3 [file Image_2.tif]

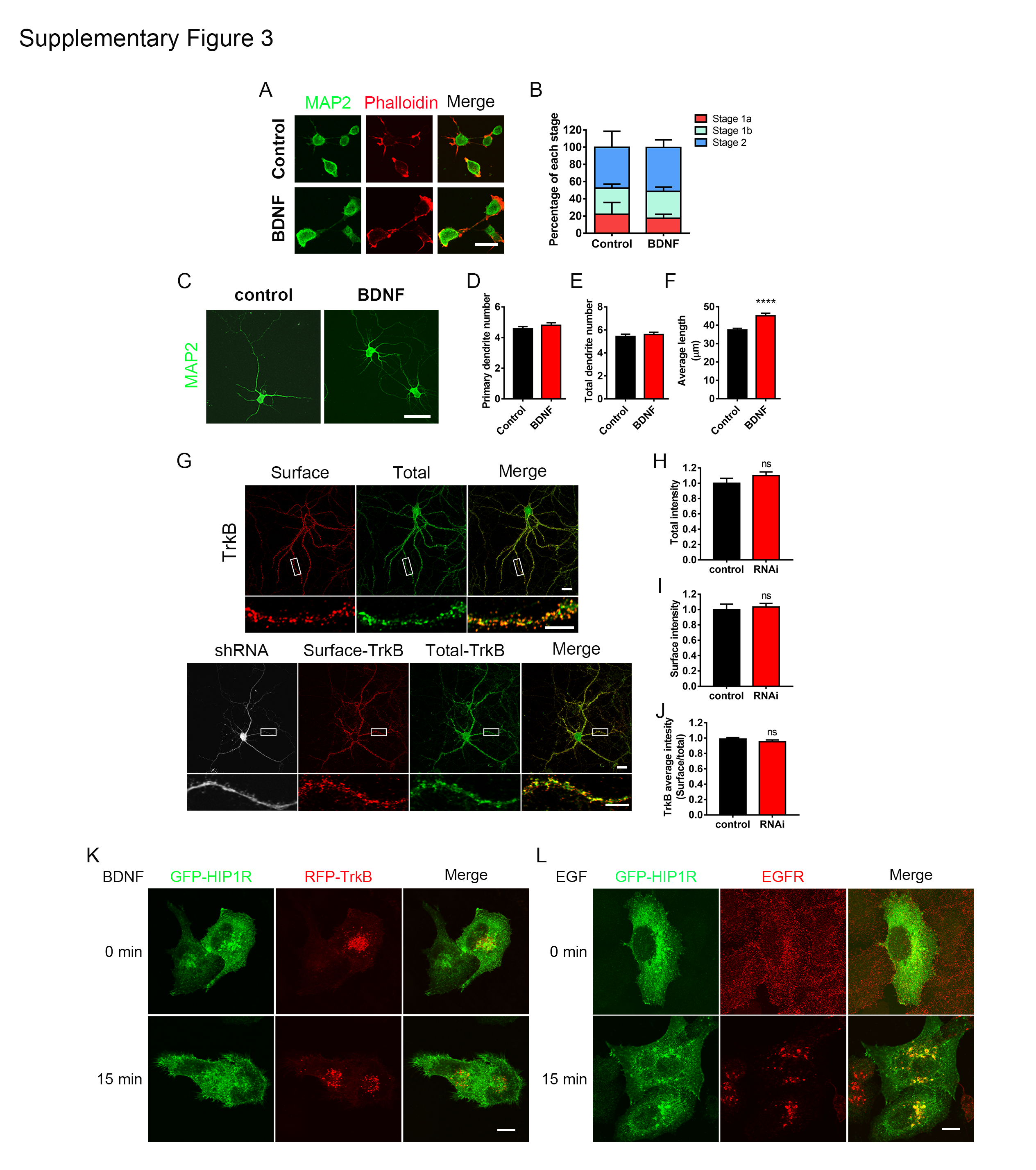

Supplement: Supplementary file 4 [file Image_3.tif]
